# Determinants of revision and functional outcome following unicompartmental knee replacement

**DOI:** 10.1016/j.joca.2014.07.006

**Published:** 2014-09

**Authors:** A.D. Liddle, A. Judge, H. Pandit, D.W. Murray

**Affiliations:** † Nuffield Department of Orthopaedics, Rheumatology and Musculoskeletal Sciences, University of Oxford, Botnar Research Centre, Windmill Road, Oxford OX3 7LD, UK; ‡ NIHR Musculoskeletal Biomedical Research Unit, University of Oxford, and MRC Lifecourse Epidemiology Unit, University of Southampton, Botnar Research Centre, Windmill Road, Oxford OX3 7LD, UK

**Keywords:** Unicompartmental knee replacement, Patient-reported outcome measures, Satisfaction, Arthroplasty

## Abstract

**Objective:**

Unicompartmental Knee Replacement (UKR) has important advantages over total knee replacement (TKR) but has a higher revision rate. Outcomes vary between centres, suggesting that risk factors for revision may be modifiable with changes to patient selection or operative technique. The objective of this study was to determine factors affecting revision, patient-reported outcome and satisfaction following UKR.

**Method:**

25,982 cases from three national databases were analysed. Multilevel multivariable regression models were used to examine the effect of patient and surgical factors on implant survival, patient-reported outcome and satisfaction at 6 months and 8 years following UKR.

**Results:**

Of the 25,982 cases, 3862 (14.9%) had pre-operative and 6-month Oxford Knee Scores (OKS). Eight-year survival was 89.1% (95% confidence intervals (CI) 88.3–89.9). OKS increased from 21.9 (SD 7.6) to 37.5 (SD 9.5). Age (Hazard ratio (HR) 0.96 (95% CI 0.96–0.97) per year), male gender (HR 0.86 (95% CI 0.76–0.96)), unit size (HR 0.92 (95% CI 0.86–0.97) per case up to 40 cases/year) and operating surgeon grade (HR 0.78 (95% CI 0.67–0.91) if consultant) predicted improved implant survival. Older patients (≥75 years), and those with lower deprivation levels had superior OKS and satisfaction (adjusted mean difference 0.14 (95% CI 0.09–0.20) points per year of age and 0.93 (95% CI 0.60–1.27) per quintile of deprivation). Ethnicity, anxiety and co-morbidities also affected patient-reported outcome.

**Conclusions:**

This study has identified important predictors of revision and patient-reported outcome following UKR. Older patients, who are least likely to be offered UKR, may derive the greatest benefits. Improved understanding of these factors may improve the long-term outcomes of UKR.

## Introduction

Unicompartmental Knee Replacement (UKR) comprises between 5% and 10% of all knee replacements recorded each year in national joint registries (NJRs), but up to 50% may be eligible on the basis of disease pattern^1^, ^2^, ^3^, ^4^. By only replacing the damaged parts of the knee, preserving normal structures such as the anterior cruciate ligament (ACL), UKR restores normal knee kinematics, restoring more normal knee function than is possible with total knee replacement (TKR)^5^, ^6^. Patients undergoing UKR recover more quickly, and have less perioperative morbidity and mortality compared to TKR, probably due to the reduced soft tissue disruption and blood loss in UKR^7^, ^8^, ^9^. However, surgeons are discouraged from using UKR as the reported revision rate is significantly higher than that of TKR^2^. This varies between units, due to differences in patient selection, surgical practice and threshold for revision of poorly-functioning implants. If predictive factors are identified and addressed, there is the potential to improve the revision rate of UKR to an acceptable level, allowing more patients to benefit from the advantages associated with UKR.

The factors predicting outcomes in TKR have been extensively studied^10^, ^11^, ^12^, ^13^, ^14^, ^15^, ^16^, ^17^, ^18^, ^19^. Amongst others, youth and male gender appear to predict revision^16^, whilst female gender, anxiety/depression and deprivation predispose to poor patient-reported outcomes and dissatisfaction with surgery^13^, ^17^, ^19^, ^20^. There is conflicting evidence about the importance of body mass index (BMI), with some studies suggesting that it affects outcome^11^, ^17^, and others reporting no effect^13^, ^21^. The extent to which these factors affect the outcome of UKR is uncertain.

As a result, there is great variation of practice regarding UKR, with some surgeons offering it to all patients who have suitable pathoanatomy, and others restricting access to UKR on the basis of criteria including age, activity level or BMI^22^, ^23^. The aim of this study is to identify factors predisposing to poor outcomes following UKR, using a cohort of 22,840 patients with linked records in three national databases.

## Patients and methods

### Data sources

The NJR for England, Wales and Northern Ireland contains details on all knee, hip and ankle replacements performed each year in both public and private hospitals in England, Wales and, since 2012, Northern Ireland. Since 2011, data collection has been mandatory^2^. Data recorded in the NJR includes prosthesis and operative information (including prosthesis type, approach and thromboprophylaxis use); patient information (age, gender, BMI, American Society of Anaesthesiologists (ASA) grade); surgical and unit information (including surgeon and unit caseload and public/private status)^2^. The Hospital Episode Statistics (HES) database records details of all hospital admissions in England. It covers a smaller geographical area than the NJR (excluding patients operated upon in Wales and Northern Ireland), and does not include privately-funded operations. However, it provides additional information, including detailed information on co-morbidities and deprivation. Since April 2009, Patient-Reported Outcome Measure (PROM) data has been collected on hip and knee replacements performed in public hospitals in England. Pre-operative and 6 month quality of life questionnaires (the EuroQol five domain (EQ5D)^24^) and joint-specific PROMs (for the knee, the Oxford Knee Score (OKS)^25^) are collected along with patient-reported measures of preoperative disability and post-operative satisfaction.

Using an extract of data from the NJR, all patients who underwent UKR between 2003 and August 2012 were identified (*n* = 41,986). Using unique identifiers, these patients were linked to corresponding HES records. Given the smaller population covered by HES, 25,982/41,986 NJR records could be linked (62%); these patients formed the study group. Given the substantially shorter time that the PROM database has been active, only 3862 of the 25,982 patients (14.9%) were linked to complete (i.e., pre- and post-operative) PROM records.

### Outcomes of interest

Primary outcome measures were revision, patient-reported outcome and satisfaction. Revision was defined as the removal, exchange or addition of any of the components of arthroplasty and was assessed using survival analysis techniques. Patient-reported outcome was assessed using the OKS. The OKS is a patient-completed questionnaire which assesses twelve domains of knee pain and function, each rated from zero (the worst score) to four (the best), completed using Likert scales^25^. It was examined in two ways, firstly, as a continuous score ranging 0–48^26^, and secondly, as a binary outcome representing patients whose OKS was worse after surgery than before. This method identifies patients who experience very poor outcomes but who do not (either through frailty or patient choice) receive revision surgery. Finally, satisfaction with surgery was rated on the basis of the question: “How would you describe the results of your operation?”, answered on a five-point scale from 1 (poor) to 5 (excellent).

### Exposures

Exposures included patient and surgical factors. Patient factors included demographics (age, gender, ethnicity); and health state (BMI, Charlson co-morbidity index and ASA grade). Pre-operative function was assessed using the OKS and EQ5D. The EQ5D consists of five questions (assessing mobility, self-care, ability to conduct usual activities, degree of pain/discomfort, and degree of anxiety/depression), ranging from 1 (best state) to 3 (worst state). EQ5D can be expressed as an overall index (graded from −0.594 to 1), or as ordinal responses for each category. Pre-operatively, patients rate their general health on a five-point Likert scale from very poor to excellent, and to report whether they considered themselves to suffer from a disability (yes or no).

Ethnicity (as reported by the patient) was grouped into six categories. These are: White (White British, White Irish and other); Mixed Ethnicity; Asian (Indian, Pakistani, Bangladeshi and other); Black (Caribbean, African and other); Other (including Chinese); and Not Given. This categorisation has been used in other PROM studies^27^.

Deprivation was assessed using the Index of Multiple Deprivation (IMD), a UK governmental assessment of the level of deprivation by area^28^. The IMD assesses 37 indicators, divided up into seven domains (Income, employment, health and disability, education, barriers to housing and services, living environment, and crime). On the basis of these indicators, all 32,482 areas are ranked; for this study, IMD was grouped by national quintiles, higher quintiles being less deprived.

Surgical factors included unit type (whilst all the cases in this cohort were publically funded, they may be performed in public or private hospitals, or in independent-sector treatment centres (ISTC)); unit size (number of cases performed per unit per year); and type of thromboprophylaxis (chemical or mechanical).

Categorisation of continuous variables was avoided wherever possible; the exception was made for non-linear predictors with established groupings (e.g., BMI)^29^. Where datasets were incomplete, the degree and pattern of missingness was assessed and, if appropriate, missing values were completed using multiple imputation (MI, see Statistical Appendix).

### Statistical analysis

Predictors of implant survival were examined using Cox regression. Univariable and multivariable models were fitted. Clustering by surgeon was accounted for by using cluster-robust standard errors. Sensitivity analyses included the use of Fine and Gray models to account for the competing risk of death (see Statistical Appendix)^30^. Survival analyses were censored at 8 years.

For continuous PROM outcomes, analysis of covariance (ANCOVA) was used. This allows examination of the effect of predictors on the OKS whilst accounting for baseline differences in OKS between patients^31^. Again, both univariable and multivariable models were constructed. Clustering by surgeon was accounted-for using a two-level random intercept model. As the multivariable model used data completed using MI, explained variation was assessed by calculating the adjusted *R*^*2*^ using the method described by Harel^32^.

For the binary PROM outcome (improvement/no change in OKS vs deterioration), logistic regression was used; again, univariable and multivariable models were used and clustering was accounted-for using a two-level random intercept model. Ordered logistic regression was used to examine the effect of each of the predictors on satisfaction.

### Regression diagnostics

The linearity of the effect of each exposure was assessed using two techniques. For survival analyses, fractional polynomial plots were produced to characterise the survival hazard^33^. For PROM (linear regression) analyses, scatterplots were produced and smoothed curves fitted using locally-weighted scatterplot smoothing (LOWESS). For non-linear predictors with established categories (such as BMI), categorisation was used; otherwise, linear splines were fitted to break the hazard down into linear subsections; these were entered separately into the model. Interactions were examined; in the case of a continuous–continuous interaction with a non-linear predictor, fractional polynomials were fitted and interactions detected using the user-written ‘mfpigen’ command in Stata^34^.

Collinearity was assessed by checking the variance inflation factor after running each model. Normality of residuals was assessed by plotting standardised normal probability–probability plots. Heteroskedasticity was assessed using graphical examination of residuals. All statistical analyses were performed using Stata IC v.12.1 for Windows (Stata Corp., College Station, TX).

## Results

**Fig. 1:**
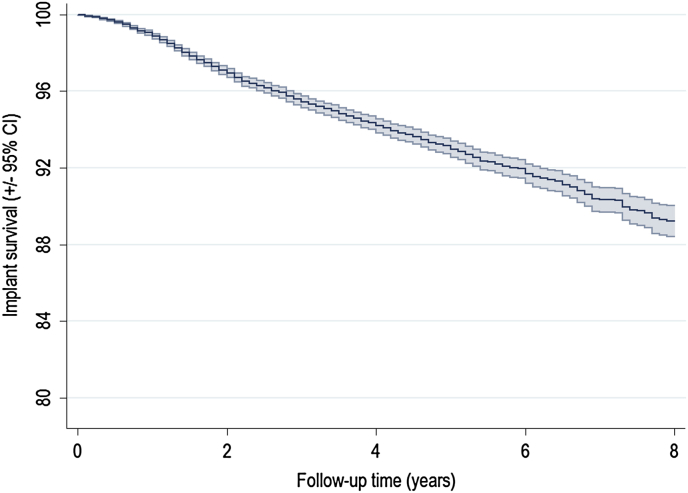
Kaplan–Meier plot demonstrating overall implant survival.

Overall implant survival was 91.8% (95% confidence intervals (CI) 91.3–92.3) at 5 years and 89.1% (95% CI 88.3–89.9) at eight. OKS increased from 21.9 (SD 7.6) points preoperatively to 37.5 (SD 9.5) points at 6 months. EQ5D index increased from a mean score of 0.480 (SD 0.290) preoperatively to 0.770 (SD 0.250) at 6 months. 84.3% of patients had good, very good or excellent levels of satisfaction. 5.5% of patients had worse OKS at 6 months than beforehand and 3.8% reported poor satisfaction. Full datasets were available for all exposures of interest except BMI which exhibited a high level of missing data. Missing BMI data were substituted using MI. The process of MI is described in detail in the statistical appendix. Descriptive statistics are displayed in Table I; outcomes are given in Table II. A Kaplan–Meier plot of overall implant survival is given in Fig. 1.

**Table I:** Baseline characteristics of the group

Number of patients (% of total)			Whole group	Patients with PROMs
			25,982 (100)	3862 (14.9)
**Variable**			**Mean (SD, Range)**	
Age			64.3 (9.7, 21.7–95.8)	64.1 (9.3, 29.2–90.9)
BMI; 13,441 (52%) missing			30.2 (5.0, 16–60)	30.0 (4.9, 16–56)
Unit size			49.4 (60.9, 1–317)	55.1 (64.2, 1–317)
**Variable**			***N* (%)**	
Gender (male)			13,547 (52.1)	2004 (51.9)
ASA grade	1		5885 (22.7)	875 (22.7)
	2		17,725 (68.2)	2695 (69.8)
	3+		2372 (9.1)	292 (7.6)
Ethnicity	Un-defined		3654 (14.1)	401 (10.4)
	White		21,506 (82.8)	3,370 (87.3)
	Mixed race		54 (0.2)	10 (0.3)
	Asian		515 (2.0)	47 (1.2)
	Black		120 (0.5)	12 (0.3)
	Other		133 (0.5)	22 (0.6)
Unit type	NHS Hospital		22,085 (85.0)	3092 (80.1)
	Private hospital		2872 (11.1)	593 (15.4)
	ISTC		1025 (4.0)	177 (4.6)
Consultant performed			22,255 (85.7)	3352 (86.8)
Mechanical thrombo-prophylaxis	TEDs		16,772 (64.6)	2607 (67.5)
	Foot pumps/calf compression		5820 (22.4)	981 (25.4)
	Other		272 (1.1)	34 (0.9)
	None		3118 (12.0)	240 (6.2)
Chemical thrombo-prophylaxis	Heparin/LMWH		15,816 (60.9)	2406 (62.3)
	Aspirin		3924 (15.1)	374 (9.7)
	Warfarin		211 (0.8)	31 (0.8)
	Direct Thrombin Inhibitor		1101 (4.2)	297 (7.7)
	Other		1927 (7.4)	507 (13.1)
	None		3003 (11.6)	247 (6.4)
Implant fixation (cemented)			23,209 (90.0)	3368 (87.7)
Charlson co-morbidity index	None		20,865 (80.3)	3038 (78.7)
	Mild		4298 (16.5)	715 (18.5)
	Moderate		642 (2.5)	92 (2.4)
	Severe		177 (0.7)	17 (0.4)
IMD (Quintiles)	1 (most deprived)		2951 (11.4)	364 (9.4)
	2		4361 (16.8)	592 (15.3)
	3		5791 (22.3)	836 (21.7)
	4		6179 (23.8)	971 (25.1)
	5 (least deprived)		6700 (25.8)	1099 (28.5)
Pre-operative OKS			21.9 (7.6, 0–43)	21.9 (7.6, 0–43)
Pre-operative EuroQol 5 domain score (EQ5D)			0.480 (0.290, −0.426 to 1)	0.480 (0.290, −0.426 to 1)
Pre-operative EQ5D anxiety/depression		None	2331 (68.0)	2331 (68.0)
		Moderate	1002 (29.2)	1002 (29.2)
		Extreme	96 (2.8)	96 (2.8)
Self-reported health		Excellent	133 (4.9)	133 (4.9)
		Very good	894 (33.0)	894 (33.0)
		Good	1209 (44.7)	1209 (44.7)
		Fair	413 (15.3)	413 (15.3)
		Poor	58 (2.1)	58 (2.1)
Self-reported disability			1347 (41.2)	1347 (41.2)

TEDS – Thromboembolic Deterrent Stockings; LMWH – Low Molecular Weight Heparin. Self-reported health and disability are additional questions on the pre-op PROM questionnaire and are not part of EQ5D.

**Table II:** Overall outcomes (patient demographics and pre-operative scores given in Table I)

Outcome		
8-year implant survival (95% CI)		89.1 (88.3–89.9)
6-month OKS, mean (SD)		37.5 (9.5)
6-month EQ5D, mean (SD)		0.770 (0.250)
Improvement, *N* (%)	Better	3,204 (93.4)
	Same	37 (1.1)
	Worse	188 (5.48)
Satisfaction, *N* (%).	Excellent	930 (27.1)
	Very Good	1223 (35.7)
	Good	725 (21.1)
	Fair	410 (12.0)
	Poor	128 (3.7)
	Not given	13 (0.4)

### Regression diagnostics

No evidence of collinearity was demonstrated for any of the models. The distribution of residuals was not demonstrated to deviate significantly from normality. Heteroskedasticity was demonstrated in the continuous OKS comparison; robust standard errors were employed with the sandwich variance estimator. Linearity of predictors is considered in the following sections.

### Factors affecting implant survival (Table III)

Implant survival was better in older people, men, and those with no co-morbidities (compared to those with moderate co-morbidities). Revision was less likely if the procedure was performed in a larger unit and if it was performed by a consultant rather than a trainee. The effect of unit size was non-linear, reducing markedly up to 40 cases/year before reaching a plateau. There was a trend towards inferior survival in ISTC compared to NHS hospitals, and with higher levels of deprivation, but these had only borderline significance. Neither BMI, ethnicity, nor thromboprophylaxis had any effect on implant survival. Survival was equivalent for cemented and cementless UKR.

**Table III:** Univariable and multivariable survival models. HR are given for all-cause revision up to 8 years following surgery

Variable			Univariable		Multivariable	
			HR (±95 CI)	Significance	HR (±95 CI)	Significance
Age (linear, per year)			0.96 (0.96–0.97)	<0.01	0.96 (0.96–0.97)	<0.01
Gender (male)			0.83 (0.74–0.94)	<0.01	0.86 (0.76–0.96)	0.01
ASA (per one grade increase)			0.94 (0.85–1.05)	0.27	1.06 (0.95–1.20)	0.30
BMI (per unit, *n* = 13,501)			1.02 (1.00–1.04)	0.05	1.01 (0.98–1.03)	0.59
Ethnic group – white			Reference			
un-defined			0.86 (0.72–1.02)	0.09	0.86 (0.72–1.02)	0.08
Mixed race			0.42 (0.07–2.65)	0.36	0.34 (0.05–2.24)	0.26
Asian			0.94 (0.60–1.46)	0.77	0.81 (0.52–1.25)	0.34
Black			1.39 (0.71–2.72)	0.33	1.02 (0.50–2.06)	0.96
Other			0.39 (0.09–1.61)	0.19	0.39 (0.09–1.61)	0.19
Unit type – NHS hospital			Reference			
Independent hospital			1.07 (0.88–1.30)	0.51	1.07 (0.87–1.30)	0.53
ISTC			1.10 (0.78–1.55)	0.59	1.43 (0.99–2.05)	0.05
Consultant performed			0.91 (0.77–1.07)	0.24	0.78 (0.67–0.91)	<0.01
Mechanical thrombo-prophylaxis	TEDS		Reference			
	Foot pumps		1.02 (0.84–1.23)	0.87	0.98 (0.81–1.19)	0.82
	Other		1.32 (0.80–2.18)	0.27	1.24 (0.77–2.01)	0.37
	None		1.04 (0.85–1.27)	0.70	1.01 (0.83–1.22)	0.94
Chemical thrombo-prophylaxis	Heparin/LMWH		Reference			
	Aspirin		0.98 (0.79–1.22)	0.85	1.11 (0.90–1.37)	0.34
	Warfarin		0.61 (0.30–1.26)	0.18	0.71 (0.35–1.44)	0.34
	DTI		0.56 (0.30–1.04)	0.07	0.55 (0.30–1.01)	0.06
	Other		0.86 (0.63–1.18)	0.36	0.81 (0.59–1.11)	0.19
	None		1.15 (0.93–1.43)	0.20	1.22 (0.98–1.51)	0.08
Fixation			0.99 (0.77–1.26)	0.91	1.02 (0.79–1.31)	0.89
Charlson – No comorbidities			Reference			
Mild comorbidities			1.09 (0.94–1.27)	0.26	1.12 (0.96–1.30)	0.16
Moderate comorbidities			1.36 (0.95–1.95)	0.09	1.49 (1.04–2.12)	0.03
Severe comorbidities			1.21 (0.62–2.35)	0.58	1.40 (0.72–2.72)	0.33
IMD (per quintile)			0.91 (0.87–0.96)	<0.01	0.96 (0.91–1.00)	0.07
Unit size (per ten cases)		Up to 40 cases/yr	0.97 (0.96–0.99)	<0.01	0.92 (0.86–0.97)	0.01
		Above 40 cases/yr			0.99 (0.97–1.01	0.32

LMWH – low molecular weight heparin; DTI – direct thrombin inhibitor.

### Factors affecting PROMs and satisfaction (Table IV)

**Fig. 2:**
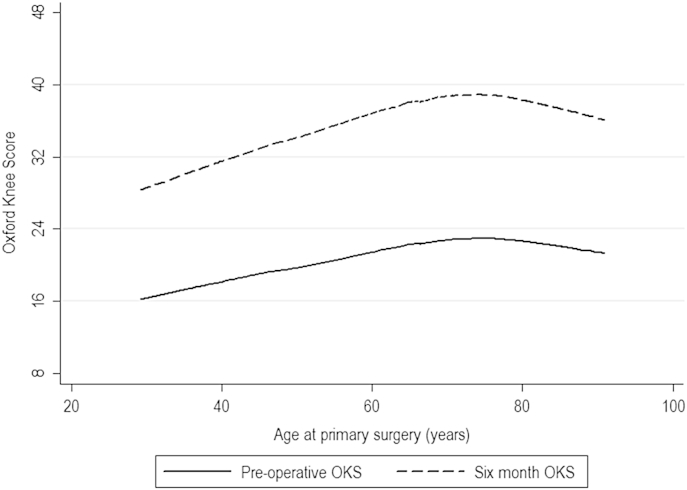
LOWESS plot of age (horizontal axis) against pre-operative (solid line) post-operative (dotted line) OKS (vertical axis) demonstrating non-linearity of the effect of age on patient-reported outcome.

Pre-operative OKS had a strong positive effect on 6-month OKS. Age strongly affected both pre-and post-operative OKS in a non-linear fashion. For both pre- and post-operative OKS, scores peaked at around 75 years of age (Fig. 2). Two linear splines were fitted, one up to 75 years and one beyond 75 years. Up to the age of 75, age had a significant positive effect on 6-month OKS (0.14 points per year, *P* < 0.001). Above 75, scores reduced but this did not reach statistical significance. These findings were adjusted for the observed baseline differences in OKS. In addition, older patients were less likely to have worse scores at 6 months than pre-operatively, and were more likely to be satisfied.

**Table IV:** PROM models

Variable				OKS model – continuous		OKS model – binary		Satisfaction	
				Adjusted mean difference (±95 CI)	Sig.	Odds ratio (±95 CI)	Sig.	Odds ratio (±95 CI)	Sig.
Age (per year)	18–75			0.14 (0.09–0.20)	<0.001	0.96 (0.94–0.98)	<0.001	1.02 (1.01–1.03)	<0.001
	≥75			−0.18 (−0.63 to 0.07)	0.162				
Gender				0.19 (−0.63 to 1.02)	0.650	1.06 (0.74–1.53)	0.744	1.01 (0.86–1.18)	0.924
ASA grade				−0.53 (−1.35 to 0.29)	0.206	1.00 (0.70–1.44)	0.998	0.88 (0.77–1.02)	0.088
BMI	Normal weight			Reference		1.00 (0.96–1.05)	0.923	1.00 (0.98–1.02)	0.979
	Underweight			4.42 (−5.06 to 13.91)	0.360				
	Overweight			−0.03 (−1.47 to 1.42)	0.972				
	Obese			−1.04 (−2.50 to 0.41)	0.160				
Ethnicity	White			Reference		Reference		Reference	
	Un-defined			0.24 (−1.08 to 1.57)	0.717	0.90 (0.46–1.75)	0.750	1.05 (0.84–1.32)	0.654
	Mixed race			−0.60 (−7.33 to 6.12)	0.860	–	–	0.65 (0.32–1.33)	0.239
	Asian			−9.77 (−14.26 to −5.27)	<0.001	1.42 (0.43–4.64)	0.564	0.41 (0.21–0.79)	0.007
	Black			−10.79 (−19.19 to −2.75)	0.009	3.65 (0.68–19.56)	0.131	0.53 (0.29–0.99)	0.048
	Other			−1.92 (−6.56 to 2.72)	0.428	–	–	0.82 (0.47–1.44)	0.494
Unit type	Public Hospital			Reference		Reference		Reference	
	Private hospital			0.48 (−0.76 to 1.72)	0.448	0.78 (0.43–1.41)	0.412	1.16 (0.90–1.49)	0.265
	ISTC			1.09 (−0.91 to 3.09)	0.286	0.26 (0.03–1.99)	0.193	1.27 (0.91–1.78)	0.154
Consultant performed				0.99 (−0.25 to 2.22)	0.116	0.56 (0.35–0.91)	0.018	1.40 (1.13–1.74)	0.002
Mechanical thrombo-prophylaxis	TEDs			Reference		Reference		Reference	
	Foot pumps			0.88 (−0.22 to 1.98)	0.117	1.01 (0.65–1.58)	0.951	1.10 (0.89–1.36)	0.362
	Other			−1.29 (−7.22 to 4.63)	0.668	0.74 (0.09–5.86)	0.773	0.71 (0.35–1.46)	0.358
	None			−1.48 (−3.37 to 0.41)	0.125	1.31 (0.68–2.53)	0.426	0.89 (0.67–1.18)	0.423
Chemical thrombo-prophylaxis	Heparin/LMWH			Reference		Reference		Reference	
	Aspirin			−1.61 (−2.54 to 0.25)	0.108	1.78 (1.02–3.12)	0.043	0.75 (0.55–1.01)	0.058
	Warfarin			−3.18 (−9.92 to 3.57)	0.356	1.17 (0.23–6.01)	0.854	1.01 (0.41–2.45)	0.988
	DTI			0.40 (−1.19 to 1.99)	0.624	0.80 (0.36–1.77)	0.584	0.82 (0.63–1.05)	0.120
	Other			−0.44 (−1.69 to 0.81)	0.486	0.77 (0.42–1.38)	0.375	0.93 (0.74–1.16)	0.509
	None			0.93 (−1.04 to 2.90)	0.356	0.83 (0.36–1.92)	0.663	1.00 (0.72–1.37)	0.985
Implant fixation				−0.56 (−1.78 to 0.65)	0.365	1.21 (0.67–2.18)	0.530	0.81 (0.65–1.02)	0.068
Charlson co-morbidity index	None			Reference		Reference		Reference	
	Mild			−0.71 (−1.79 to 0.37)	0.198	1.01 (0.63–1.61)	0.962	1.07 (0.88–1.29)	0.498
	Moderate			0.41 (−2.18 to 3.00)	0.756	2.95 (1.23–7.05)	0.015	1.21 (0.66–2.22)	0.540
	Severe			5.86 (−0.81 to 12.53)	0.085	1.72 (0.20–15.01)	0.624	2.19 (0.77–6.19)	0.140
IMD (Quintiles)				0.93 (0.60–1.27)	<0.001	0.76 (0.66–0.88)	<0.001	1.07 (1.01–1.15)	0.029
Unit size (per 10 cases)		Up to 40 cases		0.32 (−0.05 to 0.70)	0.088	0.98 (0.95–1.02)	0.354	1.01 (1.00–1.03)	0.032
		Above 40 cases		0.00 (−0.07 to 0.12	0.570				
Pre-operative OKS				0.24 (0.18 to 0.30)	<0.001	1.10 (1.07–1.13)	<0.001	1.00 (0.99–1.01)	0.807
Pre-operative EQ5D anxiety/depression			None	Reference		Reference		Reference	
			Moderate	−0.60 (−1.57 to 0.36)	0.221	1.29 (0.86–1.94)	0.220	0.97 (0.81–1.15)	0.708
			Extreme	−3.32 (−6.16 to −0.47)	0.023	1.63 (0.52–5.11)	0.404	0.92 (0.55–1.54)	0.752
Self-reported health				−1.70 (−2.24 to −1.16)	<0.001	1.30 (1.03–1.65)	0.027	0.58 (0.52–0.65)	<0.001
Self-reported disability				−2.33 (−3.24 to 1.41)	<0.001	1.92 (1.30–2.86)	<0.001	0.82 (0.70–0.96)	0.012

Adjusted mean differences are calculated in the multivariable linear regression models; positive figures demonstrate that the variable is associated with improved outcomes, negative figures demonstrate that the variable has a negative effect on post-operative OKS. TEDs – Thromboembolic Deterrent Stockings; LMWH – Low Molecular Weight Heparin; DTI – Direct Thrombin Inhibitor; OKS – Oxford Knee Score; EQ5D – EuroQol 5 Domain Score. BMI is categorised for the continuous PROM outcome as it predicts outcome in a non-linear fashion. Both satisfaction and the binary PROM outcome are predicted in a linear fashion and an overall Odds Ratio is given.

**Fig. 3:**
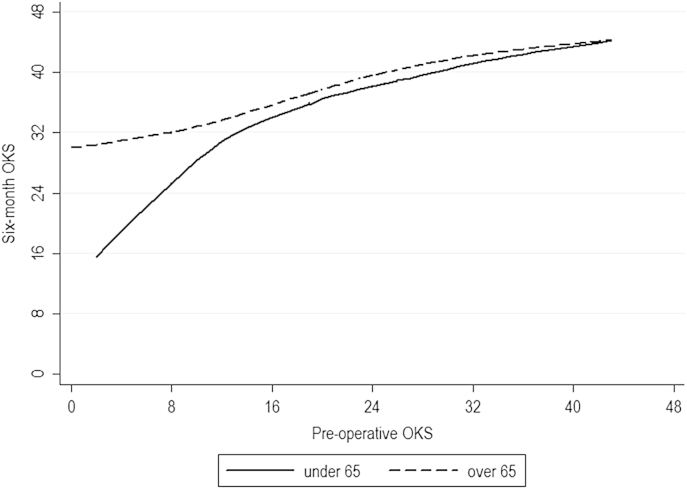
LOWESS curves of pre-operative (horizontal axis) against 6-month OKS (vertical axis), stratified by age. Whilst all groups improve substantially, young patients with very poor pre-operative scores are demonstrated to have a poorer level of improvement when compared with other patient groups.

An interaction was detected between the effects of age and pre-operative OKS level. Young patients with very poor preoperative scores appeared to fare particularly poorly: whilst poorly-functioning patients (i.e., those with a preoperative OKS ≤15) above the age of 65 can expect to gain 22.5 points on their preoperative OKS (95% CI 21.3–23.8), those below the age of 65 can only expect to gain 18.8 points (95% CI 17.7–19.8). The difference between patients above and below the age of 65 disappears for higher pre-operative OKS values (Fig. 3). No other interactions were detected.

To explore this further, the model was stratified into patients with poor pre-operative OKS (≤15) and those with better pre-operative OKS (>15); the results of these analyses are given in the statistical appendix. The stratification was not observed to affect the effect sizes or significance levels for any of the other variables studied and the non-stratified model is presented (Table IV).

Ethnicity appeared to affect outcome, with Black and Asian patients reporting significantly worse OKS and satisfaction. Patients were less likely to deteriorate and more likely to be satisfied if their operation was performed by a consultant rather than a trainee; whilst there was a trend to higher overall scores in consultant-performed cases, this was not statistically significant. Unit size did not have as great an effect on patient-reported outcome as it did on implant survival. The effect of unit size on 6-month OKS was again non-linear; OKS improved by 0.32 points for every ten cases up to 40, before becoming essentially constant above 40 cases. Only borderline significance was achieved in either section. There was a small but statistically significant increase in satisfaction if the case was performed in a higher-volume unit.

Those with co-morbidities (measured by the Charlson index) were more likely to have worse scores at 6 months than pre-operatively; patients who reported poor health prior to surgery or who described themselves as having a disability, had worse outcomes by all three metrics. Overall, thromboprophylaxis did not exert a great effect on patient-reported outcome, with the exception of patients taking aspirin, who were more likely to report deterioration in their OKS, as well as having a trend to lower overall OKS and satisfaction.

Better pre-operative OKS predicted better post-operative OKS, but patients with higher pre-op scores had a higher chance of having a worse 6 month score than they had beforehand. Very few patients underwent UKR with an OKS of more than 35 points preoperatively, but these patients (*n* = 120) had a 15.8% chance of being worse post-operatively than preoperatively; the chance of deterioration was only 5.1% in patients with a preoperative OKS of 35 points or fewer. Patients with extreme anxiety or depression (as measured using the EQ5D) had significantly worse scores overall compared to those with no anxiety/depression, but were no more likely to deteriorate or to be dissatisfied. Neither BMI, ASA grade, unit type, nor implant fixation had any effect on outcome by any measure.

## Discussion

This study has demonstrated UKR to be a highly effective intervention in relieving the symptoms of end-stage osteoarthritis. However, important risk factors for failure, in terms of patient selection and surgical practice, have been identified. Whilst there is a large body of literature exploring these factors in TKR, this is the most comprehensive study of this type in UKR.

As in previous TKR studies, pre-operative PROMs are observed to strongly predict post-operative PROMs^13^, ^18^. However, patients with severe preoperative pre-operative disease are no less satisfied following surgery despite reporting poorer PROMs. Patients with higher preoperative PROMs are also at higher risk of being worse-off following surgery than they were beforehand. This may be an issue of measurement: patients with high preoperative PROMs may make gains undetectable by the OKS^35^. This is supported by the finding that 6-month OKS and satisfaction are just as great with patients with the highest pre-operative OKS as those with lower values. However, caution should be exercised when offering any form of arthroplasty to patients with very high pre-operative function.

Age has a positive effect on outcome by each metric. Whilst OKS deteriorates with age in the general population^36^, in this cohort, young patients undergoing UKR tend to have lower baseline OKS levels than older patients, which may represent a higher threshold for offering arthroplasty to younger patients. However, even following adjustment for these differences, older patients derive the greatest benefit from UKR, and have lower revision rates than younger patients. These findings, together with the lower rates of perioperative morbidity and mortality associated with UKR^7^, ^8^, suggest that older patients fare particularly well with UKR. This is contrary to current practice: UKR comprises nearly 20% of all knee replacements performed in patients in their fifties, but only 5% in octogenarians^2^. Of particular concern would appear to be younger patients with very poor pre-operative PROMs, who appear to fare particularly poorly in terms of OKS gain.

In this cohort, more men than women undergo UKR: 58% of TKRs are performed in women, whilst the equivalent figure is only 46% in UKR^2^. Whilst men and women achieve similar PROMs and satisfaction levels, there is a small but statistically significant difference in 8-year survival, with men less likely to require revision. This contrasts with TKR, where male gender is an established risk factor for revision, despite men reporting better PROMs^10^, ^13^, ^15^, ^16^. The effect in TKR may be due to a higher infection rate in men; the relative rarity of infection following UKR may account for this difference^2^. It is unclear whether the effect of gender on survival in UKR is due to the fact that women are generally smaller than men (and hence the implants used are smaller and implantation is more technically demanding), or to some factor intrinsic to female gender. One reason may be the higher incidence of inflammatory arthropathies in women; UKRs implanted in patients with undiagnosed inflammatory arthritis are at higher risk of revision secondary to disease progression. However, given the small margin of difference in survival, and the equivalent PROMs, this study does not provide evidence for restriction of access to UKR on the basis of gender.

This study provides no evidence for restricting UKR on the basis of any of the other patient factors examined. Neither BMI nor ASA score are demonstrated to influence outcome. Whilst deprivation, morbidity, and anxiety/depression pre-dispose to a poor outcome (in terms of implant survival, PROMs and satisfaction), all are also established risk factors for poor outcomes following TKR^13^, ^14^, ^19^, ^37^. Likewise, whilst black and Asian patients have poorer outcomes in terms of PROMs and satisfaction, similar differences are observed TKR^12^. The reasons for these differences are complicated and may relate to differences in social support, pain perception and access to arthroplasty^38^. Black and Asian patients may present later in the disease process and therefore have more severe preoperative disease^27^. In this study, Black and Asian patients had significantly worse preoperative OKS than white patients. In addition, ethnicity may affect disease pattern, which may affect suitability for UKR^4^. The conclusions that can be drawn from this study in this regard are limited by the small number of patients in the ethnic groups which demonstrate significantly poorer outcomes (Black and Asian, which comprise 2.5% of the study population collectively).

Patients have better outcomes if a consultant rather than a trainee performs their UKR. Revision rates are lower, and satisfaction rates higher, in high-volume units (supporting previous studies of both TKR and UKR^39^, ^40^, ^41^). There is a large effect up to 40 cases per year, but there is a plateau above this level. This may represent improved surgical or post-surgical care in units with greater experience of UKR, or it may simply reflect the surgeons in the unit performing a greater number of cases each and hence gaining greater experience and expertise^39^, ^41^.

Whilst NJRs consistently report poorer survival in TKR when it is performed using cementless implants, this study demonstrates no such effect in UKR^2^. This supports the view that UKR may be more suitable for cementless fixation than TKR^42^. Whilst implant survival was unaffected, patients taking aspirin for thromboprophylaxis had a greater chance of deterioration with a trend to lower overall OKS and satisfaction. This may simply be an indicator of higher levels of pre-existing morbidity in patients receiving aspirin, but warrants further investigation.

### Comparison to existing studies

Whilst many studies exist which explore the factors affecting outcome following TKR, there are few equivalent studies in UKR. Pandit *et al.* reports the effect of patient factors on survival and PROMs (OKS, Knee Society Score (KSS) and Tegner activity scale) in 1000 UKRs, reporting significantly superior Tegner scores in younger and heavier patients but no differences in OKS or survival^22^. Thompson *et al.* report poorer survival in women and superior KSS scores in younger and lighter patients with no commensurate increase in revision rate^43^.

In a multi-centre study of 944 UKRs, Sebillo *et al.* reported superior survival in older patients and men, with no difference in PROMs or satisfaction for either and no effect of BMI^44^. Case series from Matharu *et al.* (459 UKRs), Kristensen *et al.* (695) and Price *et al.* (564) report no statistically significant effect of age or gender on the revision rate^45^, ^46^, ^47^. Kuipers *et al.* (437 mobile-bearing UKRs, median follow-up 2.6 years), report a higher risk of revision in patients under the age of 60 (Hazard ratio (HR) 2.2, 95% CI 1.08–4.43), but no effect of gender or BMI^48^.

### Strengths and limitations

This study represents the most comprehensive examination of risk factors for failure in UKR. The use of linked datasets has facilitated the examination of a large number of predictors. The fact that this study used an unselected registry sample with multiple surgeons and implants suggests high external validity.

Limitations relate to the potential for unmeasured confounding, particularly due to preoperative disease state^49^. The large amount of missing BMI data is a concern. However, BMI did not exert an appreciable effect on outcome in either the complete-case (see Statistical Appendix) or imputed datasets. Only short-term PROMs were available, and only on a subset of patients however, the PROM patients were similar to the overall cohort (Table I), PROMs have been demonstrated to stabilise after the first 6 months following surgery, and longer-term results have been presented in the form of revision rates^50^. PROMs by definition provide the patient's view of their result and may vary by patient characteristics even if ‘objective’ measures such as walking speed show similar results.

## Conclusions

This study has identified important surgical and patient factors which affect outcome after UKR. Of note, older patients, who are the patients least likely to be offered UKR, are those who appear to derive the greatest benefit and have the lowest revision rates. Particular caution should be exercised in younger patients with very low preoperative functional scores. No evidence has been found for the use of narrow indications for UKR in terms of BMI or ASA, and many other prognostic factors are common to both UKR and TKR. Superior outcomes have been demonstrated for patients operated upon by consultant surgeons in high-volume units, supporting the findings of previous studies which report an important effect of surgical experience in generating acceptable outcomes from UKR. The information provided by this study should inform patients and surgeons when deciding whether to proceed with UKR in cases of end-stage osteoarthritis.

## Author contributions

The corresponding author, Mr Liddle, is the guarantor and, as such, takes responsibility for the integrity of the work as a whole.

AD Liddle made substantial contributions to study conception and design, data acquisition and analysis, drafted the article and approved the final version.

A Judge made substantial contributions to study conception, design, and data analysis (as the senior statistical author), revised the article prior to submission and approved the final version.

H Pandit made substantial contributions to study design and data interpretation, revised the article prior to submission and approved the final version.

DW Murray made substantial contributions to study conception and design, extensively revised the article and approved the final version.

## Role of funding source

This study was funded by grants from the Royal College of Surgeons of England and Arthritis Research UK. Neither body played any role in the conduct of the study or the decision to publish.

## Conflicts of interest

ADL and AJ report no relevant conflicts of interest. HP has been a paid speaker for Biomet, who are manufacturers of orthopaedic implants including unicompartmental and total knee replacements. DWM receives royalties and is paid consultancy fees by Biomet. The institution receives research funding from Biomet, Stryker and Zimmer, all of whom are manufacturers of orthopaedic implants.

None of these companies were involved in the funding or conduct of this study.

## Figures and Tables

**Table:** 

Variable			Competing risks model		Cox model (as in paper)	
			SHR (±95 CI)	Significance	HR (±95 CI)	Significance
Age (linear, per year)			0.96 (0.96–0.97)	<0.01	0.96 (0.96–0.97)	<0.01
Gender (male)			0.85 (0.76–0.94)	<0.01	0.86 (0.76–0.96)	0.01
ASA (per one grade increase)			1.07 (0.96–1.18)	0.22	1.06 (0.95–1.20)	0.30
BMI (per unit, *n* = 13,501)			1.01 (0.98–1.03)	0.59	1.01 (0.98–1.03)	0.59
Ethnic group – white			Reference			
un-defined			0.86 (0.74–1.01)	0.07	0.86 (0.72–1.02)	0.08
Mixed race			0.34 (0.05–2.42)	0.28	0.34 (0.05–2.24)	0.26
Asian			0.80 (0.52–1.24)	0.32	0.81 (0.52–1.25)	0.34
Black			1.02 (0.50–2.07)	0.96	1.02 (0.50–2.06)	0.96
Other			0.39 (0.10–1.57)	0.19	0.39 (0.09–1.61)	0.19
Unit type – NHS hospital			Reference			
Independent hospital			1.06 (0.88–1.29)	0.53	1.07 (0.87–1.30)	0.53
ISTC			1.40 (0.96–2.03)	0.08	1.43 (0.99–2.05)	0.05
Consultant performed			0.78 (0.67–0.91)	<0.01	0.78 (0.67–0.91)	<0.01
Mechanical thrombo-prophylaxis	TEDS		Reference			
	Foot pumps		0.97 (0.84–1.21)	0.68	0.98 (0.81–1.19)	0.82
	Other		1.22 (0.74–1.99)	0.27	1.24 (0.77–2.01)	0.37
	None		1.00 (0.86–1.17)	0.96	1.01 (0.83–1.22)	0.94
Chemical thrombo-prophylaxis	Heparin/LMWH		Reference			
	Aspirin		1.10 (0.94–1.29)	0.22	1.11 (0.90–1.37)	0.34
	Warfarin		0.70 (0.34–1.50)	0.36	0.71 (0.35–1.44)	0.34
	DTI		0.55 (0.30–1.00)	0.05	0.55 (0.30–1.01)	0.06
	Other		0.80 (0.58–1.11)	0.18	0.81 (0.59–1.11)	0.19
	None		1.21 (1.04–1.41)	0.02	1.22 (0.98–1.51)	0.08
Fixation			1.02 (0.83–1.25)	0.87	1.02 (0.79–1.31)	0.89
Charlson – No comorbidities			Reference			
Mild comorbidities			1.13 (0.97–1.31)	0.13	1.12 (0.96–1.30)	0.16
Moderate comorbidities			1.46 (1.04–2.05)	0.03	1.49 (1.04–2.12)	0.03
Severe comorbidities			1.26 (0.63–2.53)	0.51	1.40 (0.72–2.72)	0.33
IMD (per quintile)			0.96 (0.92–1.00)	0.04	0.96 (0.91–1.00)	0.07
Unit size (per ten cases)		Up to 40 cases/yr	0.99 (0.99–1.00)	<0.01	0.92 (0.86–0.97)	0.01
		Above 40 cases/yr	1.00 (1.00–1.00)	0.13	0.99 (0.97–1.01)	0.32

Estimates from competing risks and Cox regression models for survival. SHR – Subhazard Ratio; TEDS – Thromboembolic deterrent stockings; LMWH – Low Molecular Weight Heparin; DTI – Direct Thrombin Inhibitor.

**Table:** 

Analysis		Univariable	Multivariable (complete case)	Multivariable (imputed)
Survival, hazard ratio		1.02 (1.00–1.04), *P* = 0.05	1.00 (0.99–1.02), *P* = 0.66	1.01 (0.98–1.03), *P* = 0.59
OKS (continuous), coefficient	Underweight	1.40 (−8.50 to 11.30), *P* = 0.78	4.34 (−5.14 to 13.83), *P* = 0.37	4.42 (−5.06 to 13.91), *P* = 0.36
	Normal	Reference	Reference	Reference
	Overweight	−0.03 (−1.27 to 1.21), *P* = 0.96	−0.24 (−1.47 to 1.42), *P* = 0.97	−0.03 (−1.47 to 1.42), *P* = 0.97
	Obese	−1.50 (−2.74 to −0.26), *P* = 0.02	−1.13 (−2.59 to 0.33), *P* = 0.13	−1.04 (−2.50 to 0.41), *P* = 0.16
OKS (binary), odds ratio		1.02 (0.98–1.06), *P* = 0.31	1.00 (0.95–1.04), *P* = 0.89	1.00 (0.96–1.05), *P* = 0.92
Satisfaction, odds ratio		0.97 (0.96–0.99), *P* < 0.001	1.00 (0.98–1.02), *P* = 0.99	1.00 (0.98–1.02), *P* = 0.98

## Statistical Appendix

Competing-risks survival analysis.

As a sensitivity analysis, the multivariable survival analysis conducted in the first part of the study was repeated, adjusting for the competing risk of death using the method of Fine and Gray. Only minimal differences were determined between the estimates of the two models. As such the Cox model is given in the paper. The two models are compared in the table below.

Missing data: BMI.

The NJR is the only joint registry worldwide which collects data relating to BMI. As has been noted in previous analyses of NJR data, a large proportion of the BMI data are missing. In this instance, BMI data are only available on 13,501 of the 25,982 records (52.0%); of those with PROM data, this figure is 2553 of 3862 (66.1%).

Missing BMI data were explored and appear to be missing at random. Analyses were conducted on the complete dataset with and without BMI, and then the missing data were completed using MI.

MI was performed using the MI suite of commands in Stata (in this case, using truncated regression). Ten imputed datasets were created using all other available exposure variables and the outcome variable (either the continuous OKS outcome, the continuous satisfaction outcome or the Neilson–Aalen estimator of survival, depending on the analysis). MI was performed separately for each of the comparisons studied.
